# Enantioconvergent Copper-Catalysed Difluoromethylation of Alkyl Halides

**DOI:** 10.21203/rs.3.rs-3645899/v1

**Published:** 2024-08-16

**Authors:** Wei Liu, Decai Ding, Lingfeng Yin, Andrew Poore, Yeu-Shiuan Ho, Yu-Ho Cheng, Chi-Tien Hsieh, Stephen Yachuw, Rachael Knieser, Jeanette Krause, Shiliang Tian, Mu-jeng Cheng

## Abstract

Stereochemical-controlled hydrogen bond donors play essential roles in the pharmaceutical industry. Consequently, organic molecules that bear difluoromethyl (CF2H) groups at chiral centers are emerging as pivotal components in pharmaceuticals due to their distinct hydrogenbonding property. However, a general approach for introducing CF2H groups in an enantioselective manner remained elusive. Here, we show that enantioconvergent difluoromethylation of racemic alkyl electrophiles, through alkyl radical intermediates, represents a new strategy for constructing CF2H-containing stereocenters. This strategy is enabled by using copper catalysts bound with a chiral diamine ligand bearing electron-deficient phenyl groups, and a nucleophilic difluoromethyl-zinc reagent. This method allows for the high-yield conversion of a diverse range of alkyl halides into their alkyl-CF2H analogs with excellent enantioselectivity (up to 99% e.e.). Mechanistic studies, supported by DFT calculations, revealed a route involving asymmetric difluoromethylation of alkyl radicals and crucial non-covalent interactions in the enantio-determining steps.

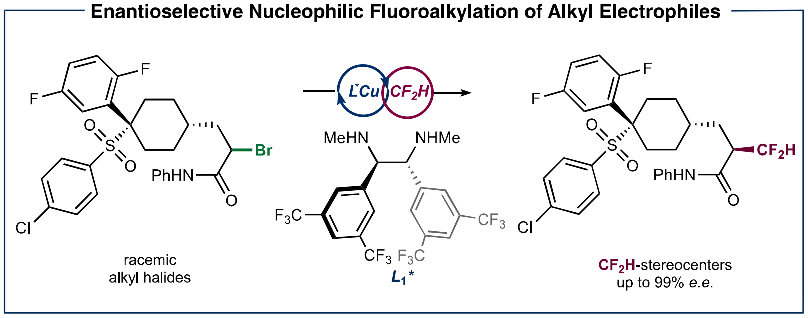

### Main Text:

The pharmaceutical industry places high importance on stereochemical-controlled hydrogen bond donors for modulating drug-target interactions. One emerging approach to enhance the properties of parent drug candidates has been to replace traditional hydrogen bond donors (*e.g.,* OH, $NH_2$, or SH) with metabolically stable analogs.[1] In this context, the difluoromethyl ($CF_2H$) groups have emerged as privileged groups in medicinal chemistry due to their unique hydrogen bond donor potential and their capacity to function as bioisosteres of OH, $NH_2$, and SH groups.[2, 3] Beyond their hydrogen-bonding capabilities, the lipophilic nature of $CF_2H$ groups and the strong dissociation energy of carbon-fluorine bonds can further modulate the membrane permeability and metabolic stability of drug molecules.[4] The significance of stereochemically-defined $CF_2H$ groups has been recently showcased by the development of two pharmaceutical agents, Inavolisib[5] and LPC-233[6] (**Fig. 1a**). The efficacy of the former has been attributed to the hydrogen-bonding ability of its $CF_2H$ group with a serine residue of phosphoinositide 3-kinase, whereas the potency of the latter molecule has been attributed to the electrostatic interaction of the fluorine atoms with a lysine residue of the enzyme LpxC.

However, efforts to fully exploit the potential of $CF_2H$ groups in medicinal chemistry were hampered by the challenges associated with their preparation; limited synthetic methods are currently available for the enantioselective introduction of $CF_2H$ groups.[7-9] Conventional approaches for constructing $CF_2H$-containing stereogenic centers relied on either the deoxyfluorination of enantioenriched aldehydes[10, 11] or the asymmetric transformation of $CF_2H$-containing prochiral molecules.[12, 13] These approaches generally suffered from either the narrow substrate scope of the fluorination reagents or the limited availability of the prochiral precursors. Recent advances in asymmetric difluoromethylation have focused on the manipulation of C(sp$^2$) electrophiles. For example, the Hu group has made progress for the asymmetric difluoromethylation of aldehydes[14] and imines[15], albeit with moderate enantioselectivity. The Jacobsen group has reported an unusual migratory geminal difluorination of styrenes for the construction of $CF_2H$-containing stereocenters at benzylic positions.[20] Shen and Mikami have recently demonstrated the difluoromethylation of allyl substrates via either $S_N2'$[16] or Michael addition[17] pathways, although these transformations yielded products with modest enantioselectivity. An alternative strategy in this theme involves the use of difluoroenoxysilanes as masked $CF_2H$ sources, as exemplified by Zhang's asymmetric difluoroalkylation of propargyl sulfonate[18] and Ma's synthesis of enantioenriched isoindolones.[19] Enantioselective difluoromethylation via the insertion of difluorocarbene has recently emerged, enabling the synthesis of specific $CF_2H$-containing moieties, including α-amino acids[20] and β-ketoesters.[21] Given the unique properties of $CF_2H$ groups, a new and more general mode of asymmetric difluoromethylation is highly desired to expand the molecular architectures containing the $CF_2H$ bioisostere.

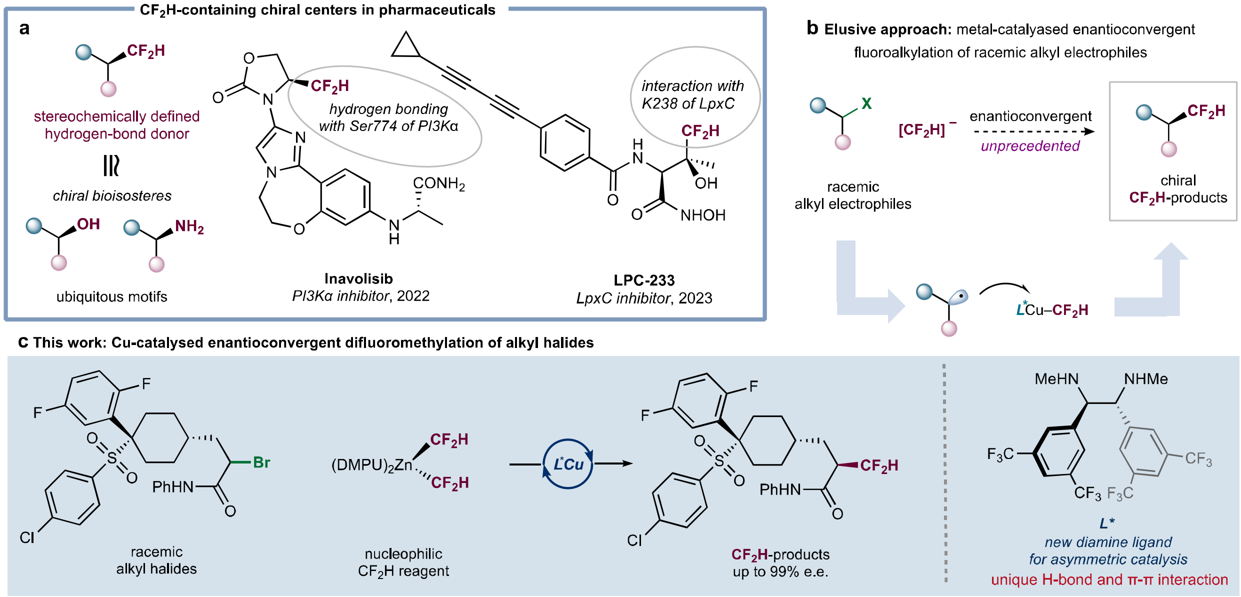

**Fig. 1 Development of Cu-catalysed enantioconvergent difluoromethylation of alkyl halides. a**. Importance of $CF_2H$-bearing chiral centers in the pharmaceutical industry. b. nucleophilic enantioconvergent fluoroalkylation as a new approach for the construction of chiral $C(sp^3)$–$CF_2H$ moieties **c.** Cu-catalysed enantioconvergent difluoromethylation alkyl halides enabled by a chiral diamine ligand.

Nucleophilic difluoromethylation of racemic $C(sp^3)$-electrophiles in an enantioconvergent manner represents one of the most mechanistically straightforward approaches to the construction of chiral $C(sp^3)$–$CF_2H$ moieties (**Fig. 1b**). Nonetheless, despite the considerable progress in the use of chiral transition-metal catalysts for the substitution of racemic alkyl electrophiles with carbon-[22-24] oxygen-[25] and nitrogen-centered nucleophiles,[26-28] enantioselective difluoromethylation via this pathway remained largely underdeveloped. Moreover, only recently have methods for transition metal-catalysed fluoroalkylation of alkyl electrophiles emerged, with a focus mainly on trifluoromethylation reactions.[29-31] This lag in the development of nucleophilic fluoroalkylation reactions is attributed to the sluggish oxidative addition of the metal-fluoroalkyl species with alkyl electrophiles and the slower reductive elimination of metal-fluoroalkyl species compared to their non-fluorinated counterparts.

Our group[32-35] and others[36] have recently demonstrated the potential of copper catalysts in facilitating the transfer of $CF_2H$ groups to aliphatic sites through alkyl radical intermediates. These catalytic methods enabled the synthesis of racemic $CF_2H$-containing products from various alkyl electrophiles. We recently questioned the feasibility of enantioselective transfer of $CF_2H$ groups to alkyl radicals when the copper catalysts are in a chiral environment. This could enable a general strategy for the construction of difluoromethylated stereocenters. We realized that several challenges should be addressed to achieve such an enantioselective transformation. First, the intrinsic instability of the [Cu–$CF_2H$] species compared to their $CF_3$ counterparts[37, 38] requires a swift single electron transfer (SET) event between this intermediate and the alkyl electrophile to prevent off-cycle decomposition. In addition, the formation of the anionic species $[Cu^I(CF_2H)_2]^-$, which is known to react with alkyl electrophiles in a racemic fashion,[39] needs to be minimized. The pinnacle challenge, however, is to enable the enantioselective transfer of $CF_2H$ groups to alkyl radicals, an endeavor that had yet to be achieved.[40, 41] In this work, we solved all the above challenges by employing an electron-deficient chiral diamine ligand (**Fig. 1c**). We disclose herein a new mode for enantioselective difluoromethylation through alkyl radical intermediates, enabling the high-yield synthesis of a diverse range of $CF_2H$-containing molecules with excellent enantioselectivity. DFT calculations demonstrate key hydrogen bonding and π-π interactions between the chiral diamine ligand and substrates.

**Results:**

We first examined the difluoromethylation of α-haloamides given the prevalence of amide groups in bioactive molecules (see Table **S1-3** for detailed optimization results). Our study revealed that the combination of copper salts and a commercially available chiral diamine ligand, **L***, catalysed the difluoromethylation of racemic α-bromo-*N*-phenylbutanamide in 85% yield and with 95% e.e. (**Fig. 2, Entry 1**). A nucleophilic difluoromethyl zinc reagent, $(DMPU)_2Zn(CF_2H)_2$, *i.e.* Vicic-Mikami reagent,[42, 43] was essential to promote this reaction. The use of $\mathbf{L}^*$, which bears electron-deficient phenyl groups, was uniquely effective for this difluoromethylation reaction. Notably, the use of $\mathbf{L}^*$ has not been reported in any reactions prior to this study. The sub-ambient temperature is necessary to achieve both high enantiocontrol and efficiency of the reactions, presumably by decelerating the decomposition pathways of the $Cu^I$–$CF_2H$ intermediate. Additionally, using solvents with higher polarity significantly decreased the enantioselectivity of products presumably due to the partial formation of the anionic $[Cu^I(CF_2H)_2]^-$ species.

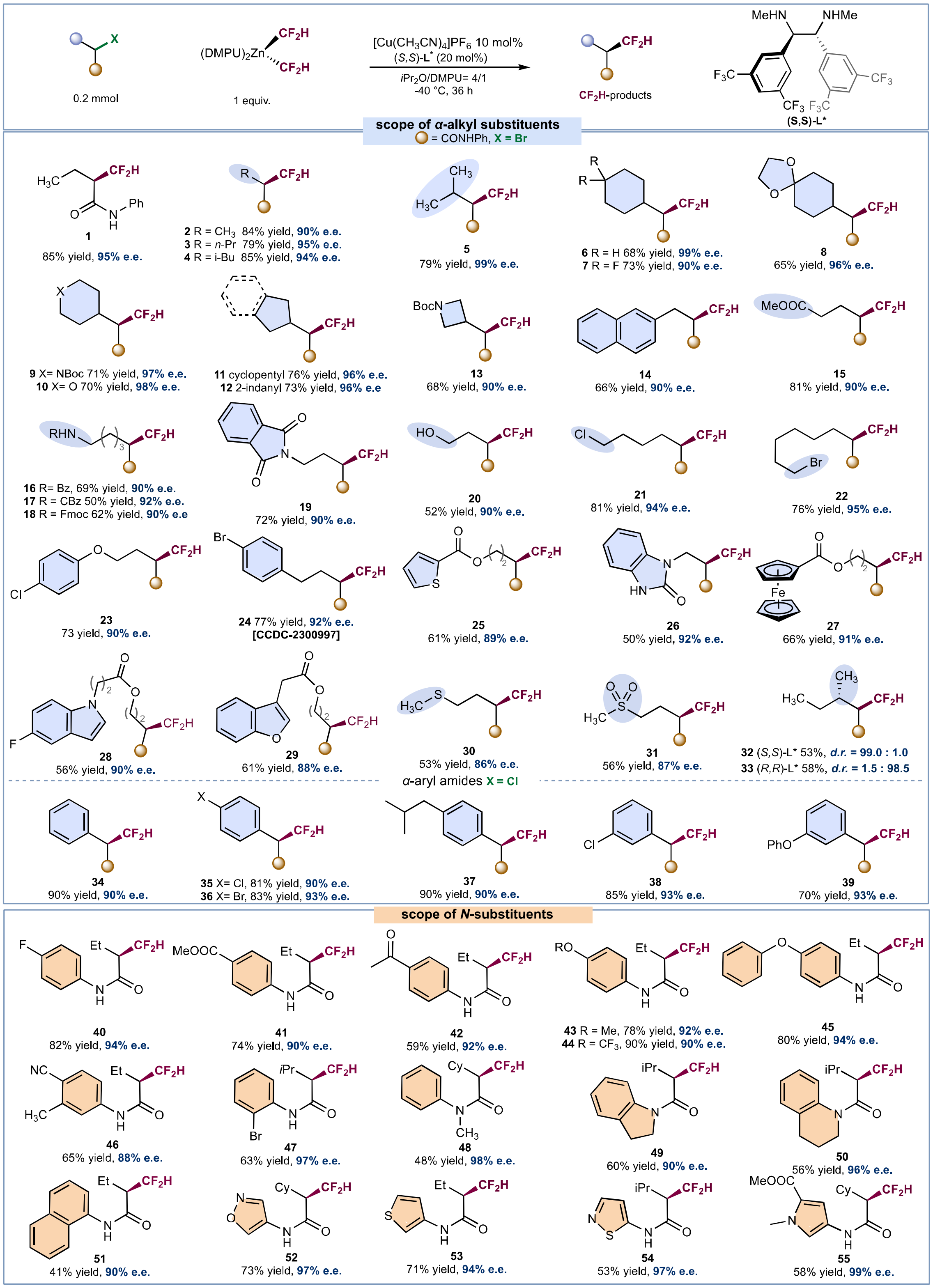

**Fig. 2. Scope for Cu-catalysed enantioconvergent difluoromethylation of alkyl halides.** Reactions were performed with 0. 2 mmol alkyl halides, 0.2 mmol $(DMPU)_2Zn(CF_2H)_2$, 0.02 mmol (10 mol%) $[Cu(CH_3CN)_4]PF_6$ and 0.04 mmol (20 mol%) **L*** in 1.5 mL $iPr_2O$/DMPU (4/1) at –40 °C for 36 h. Yields were based on isolated products. Enantiomeric excesses (e.e.) were determined by HPLC analysis on a chiral stationary phase.

We next examined the generality of this asymmetric difluoromethylation reaction. α-bromoamides with simple alkyl substituents afforded the difluoromethylated products in good yield (79–85% yield), with enantioselectivity ranging from 90–95% e.e. (**2–4**). Alkyl bromides adjacent to secondary cyclic and acyclic groups were competent, affording the desired products in good yield and with high enantioselectivity (**5-12**, 65–79% yield, 90–99% e.e.). Notably, medicinally relevant heterocycles including piperidine (**9**), tetrahydropyran (**10**), and azetidine (**13**) were compatible with this protocol. In addition, a substrate that contained the bromide at a homobenzylic position, which was prone to $\beta$-hydrogen elimination, could afford its difluoromethylated product **14** in 66% yield and with 90% e.e. Various functional groups including ester (**15**), amide (**16**), carbamate (**17–18**), imide (**19**), and even unprotected alcohol (**20**) could be well tolerated (52–81% yield, 90–92% e.e.).

Substrates containing alkyl and aryl halides (bromides and chlorides) could be successfully difluoromethylated in good yield and with high enantioselectivity without affecting these halogen atoms (**21–24**, 73–81% yield, 90–95% e.e.). Heterocycles including thiophene (**25**), benzimidazolinone (**26**), ferrocene (**27**), indole (**28**), and benzofuran (**29**) were well tolerated under the mild reaction conditions, affording the difluoromethylated products with high enantioselectivity (50–66% yield, 88–92% e.e.). Substrates that contain strong coordinating functional groups including thioether (**30**) and sulfone (**31**) were converted to the desired products with slightly diminished enantioselectivity (86% and 87% e.e.). Moreover, stereoselectivity issues were probed in the reactions of a leucine-derived substrate (**32** and **33**). Catalysts with each of the enantiomers of ligand $\mathbf{L}^*$ were tested, and both led to products with excellent diastereoselectivity (d.r. ≥ 98.5:1.5), reflecting high levels of catalyst- rather than substrate-controlled stereoselectivity. Additionally, α-aryl-α-chloro-substituted amides could serve as suitable electrophiles, affording products that contain $CF_2H$ groups at benzylic center in high yield and with good enantioselectivity (**34–39**, 70-90% yield, 90-93% e.e.). The absolute configuration of the chiral molecules were determined by the X-ray crystallography of compound **24** (CCDC-2300997, **Fig. S1**), which contained a chiral center of *R* configuration.

In addition to *N*-phenyl-amide, we evaluated the compatibility of other *N*-aryl and *N*-heteroaryl groups with this asymmetric difluoromethylation reaction. Gratifyingly, electron-rich and electron-deficient substituents at *para, meta, or ortho* positions of the phenyl groups have little effect on the enantioselectivity of the difluoromethylated products (**40–47**, 59–90% yield, 88–97% e.e.). Additionally, substrates that contain tertiary amide groups could afford desired products with high enantioselectivity (**48–50**, 48–60% yield, 90–98% e.e.). These results rule out the involvement of aziridinone, an intermediate proposed in an asymmetric phenoxylation system.[25] Finally, a diverse range of *N*-heteroaryl groups, such as naphthalene (**51**), isoxazole (**52**), thiophene (**53**), isothiazole (**54**), and pyrrole (**55**) were compatible with this difluoromethylation protocol, furnishing the products in high enantioselectivity (41–73% yield, 90–99% e.e.).

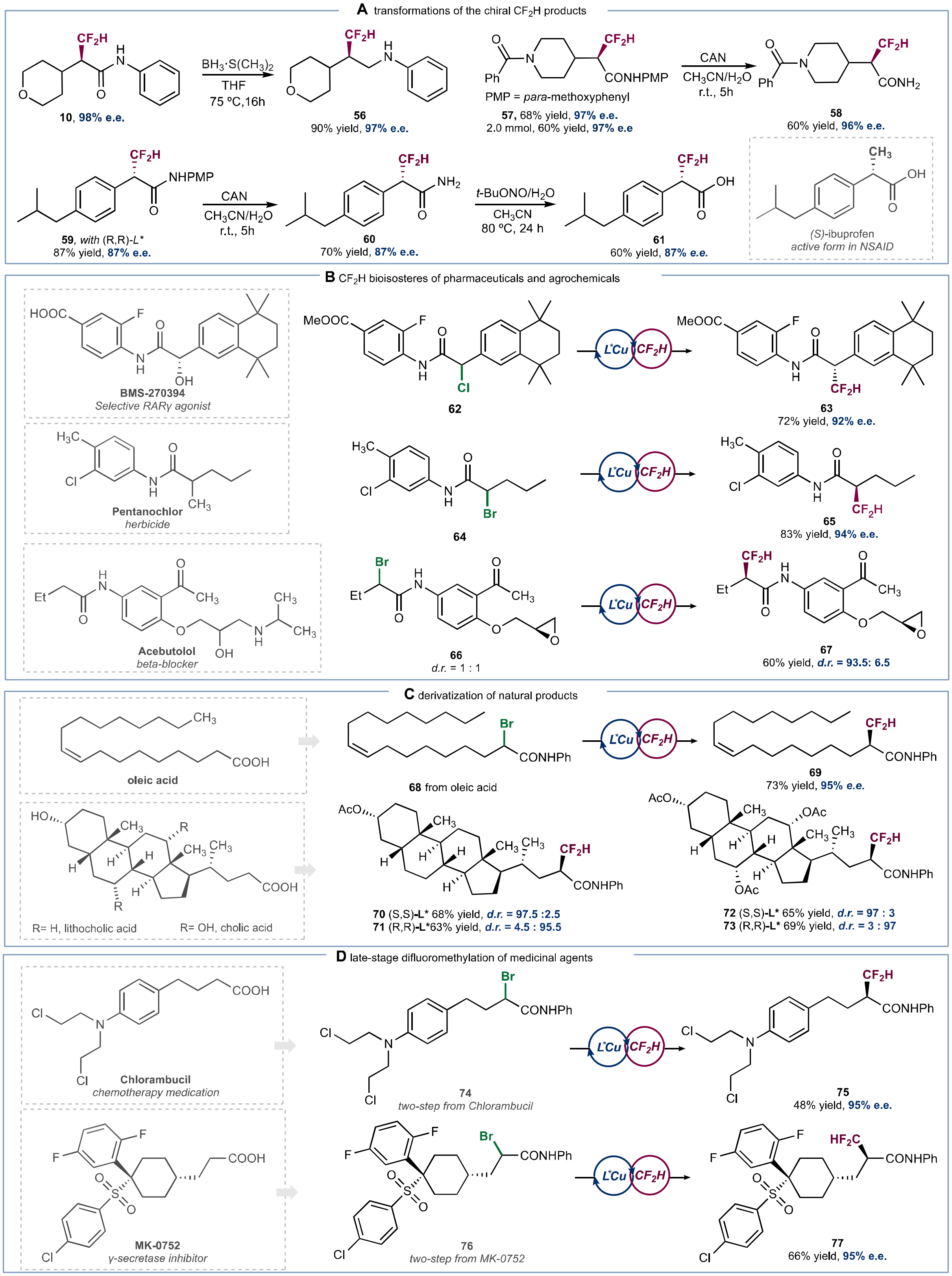

**Fig. 3 Synthetic applications of enantioconvergent difluoromethylation reaction. A.** synthetic transformation of enantiopure $CF_2H$ products; **B.** synthesis of analogs of pharmaceuticals and agrochemicals; **C**. late-stage asymmetric difluoromethylation of natural products; **D.** late-stage difluoromethylation of medicinal agents

### Synthetic application

We further showcased that this asymmetric difluoromethylation protocol could facilitate the efficient synthesis of $CF_2H$ analogs of molecules with pharmaceutical significance (**Fig. 3).** First, the diverse reactivity of amide groups allowed for the synthesis of different $CF_2H$-containing molecules without affecting the chiral centers (**Fig. 3A**). For example, the reduction of compound **10 (**98% e.e.) with borane provided the *β*-difluoromethylamine **56** in 90% yield with 97% e.e. In addition, the *N-para*-methoxy phenyl amide group in compound **57** (97% e.e.) could be removed via the cerium ammonium nitrate (CAN) oxidation to afford a primary amide **58** in 60% yield with 96% e.e. The scalability of this difluoromethylation protocol has also been demonstrated by the synthesis of compound **57** at a half-gram scale (60% yield, 97% e.e.). Moreover, recognizing the benefits of fluorinated analogs of nonsteroidal anti-inflammatory drugs (NSAIDs), we synthesized the difluoromethylated counterpart of (*S*)-ibuprofen (**Fig. 3A**). The $CF_2H$ amide **59** was formed in 87 % yield and with 87% e.e. from the corresponding benzylic chloride. A two-step approach allows for the conversion of **59** to the $CF_2H$ analog of (*S*)-ibuprofen (**61**, 87% e.e.) without compromising the enantioselectivity.

Given the widespread presence of amide functionalities in pharmaceuticals and agrochemicals, our approach offered an invaluable way for the rapid synthesis of their enantioenriched fluorinated bioisosteres (**Fig. 3B**). BMS-270394, a selective agonist for the human retinoic acid receptor (hRAR), possesses a hydroxyl moiety that binds to the methionine sulfur atom within the protein's active site.[44, 45] This hydroxyl group plays a pivotal role in the activity of BMS-270394, while its enantiomer remains inactive. Recognizing the potential of $CF_2H$ groups as bioisosteres of OH groups, we successfully transformed the benzyl chloride precursor (**62**) into the difluoromethylated analog of BMS-270394 (**63**) in 72% yield and with 92% e.e.

Moreover, given the escalating relevance of fluorinated herbicides and the increasing frequency of chiral centers in these molecules,[46] we applied this asymmetric difluoromethylation protocol to the synthesis of a chiral $CF_2H$ analog of pentanochlor, a pre- and post-emergence herbicide. Thus, the treatment of the alkyl bromide **64** under the standard difluoromethylation conditions allowed for the synthesis of **65** in 83% yield and with 94% e.e. Furthermore, this protocol enabled the synthesis of a precursor to the $CF_2H$ analog of acebutolol,[47] a beta-blocker for the treatment of high blood pressure (**67**, 60% yield, *d.r.* = 93.5 : 6.5). Notably, the tolerance of a reactive epoxide group further demonstrated the mild conditions of this Cu-catalysed protocol.

We have extended this protocol to the late-stage difluoromethylation of natural products (**Fig. 3C**). A facile α-bromination followed by the amide bond formation of oleic acid affords the corresponding alkyl bromide (**68**), which could be converted to its difluoromethylated product (**69**) with high enantioselectivity (73% yield, 95% e.e.). Similarly, two steroid derivatives, cholic acid and lithocholic acid (in their acetate forms), were converted to their difluoromethylated analogs with excellent control of stereochemistry (**70–73**, 63–69% yield, d.r. > 20:1) The high diastereoselectivity with either enantiomer of the diamine catalyst **L*** further highlights the catalyst-controlled selectivity of this difluoromethylation protocol.

Finally, this late-stage asymmetric difluoromethylation reaction protocol could be applied to the functionalization of pharmaceutical agents (**Fig. 3D**). Thus, a chemotherapy medication chlorambucil[48] and a γ-secretase inhibitor MK-0752[49] could be readily converted to their corresponding alkyl bromides (**74** and **76**). Difluoromethylation of these two alkyl bromides under the standard conditions afforded their $CF_2H$ products (**75** and **77**) in good yield and with high enantioselectivity (48 and 66% yield, respectively, 95% e.e.).

**Mechanistic studies**

These results shown herein represent a rare example of highly enantioconvergent fluoroalkylation of alkyl electrophiles. Detailed mechanistic insights into these catalytic reactions should inspire the future development of asymmetric fluoroalkylation reactions. The radical nature of this protocol was confirmed by the difluoromethylation of a cyclopropyl-containing substrate (**78**), which afforded the only ring-opening products (**79** and **80**), whereas the unrearranged product (**81**) was not observed (**Fig. 4a**). In addition, we employed a radical trap DMPO (5,5-dimethyl-1-pyrroline *N*-oxide) to trap the transient alkyl radicals formed in the reaction mixture. The addition of DMPO to the reaction mixtures containing $[Cu(MeCN)_4]PF_6$, **L***, and an alkyl bromide **82**, with or without $(DMPU)_2Zn(CF_2H)_2$, consistently revealed the formation of the trapping product **83**, which was detected by electron paramagnetic resonance (EPR) spectroscopy. This observation further suggests that the reaction mechanism involves a SET step from the copper(I) species, leading to the generation of an alkyl radical (**Fig. 4a)**. EPR simulations indicated hyperfine coupling to both nitrogen and hydrogen nuclei, with hyperfine constants $A_N$ = 6.5 G, 6.5 G, 34 G and $A_H$ = 23 G, 21 G, 23 G. Notably, the average hyperfine splitting values at low temperature align closely with those previously reported for DMPO radicals at room temperature.[24]

We further applied EPR spectroscopy to investigate the potential formation of $Cu^{II}$ intermediates through SET (**Fig. S2**). A solution of diamagnetic $[Cu(CH_3CN)_4]PF_6$ with the chiral diamine ligand **L*** exhibited a negligible EPR signal from the cavity. The addition of an alkyl bromide to this solution led to the emergence of an EPR signal with hyperfine splitting characteristics of a $Cu^{II}$ species, implying the formation of a $Cu^{II}$ intermediate in the reaction pathway. In analogous experiments, we introduced $(DMPU)_2Zn(CF_2H)_2$ to the reaction mixture. Interestingly, the resulting EPR spectra were silent, suggesting the rapid reaction between the transient alkyl radicals with the paramagnetic $[Cu^{II}–CF_2H]$ species.

To shed light on the enantioconvergent process of this reaction, we conducted difluoromethylation of an enantiomerically pure alkyl bromide **84 (Fig. 4b)**. Under standard reaction conditions, both enantiomers were converted to the same difluoromethylated product **2** with an identical e.e. value and in similar yield. Moreover, analysis of the unreacted alkyl bromides throughout the reaction revealed that no racemization occurred during the difluoromethylation reactions. These results are consistent with a stereoablative enantioconvergent process[50] rather than a simple kinetic resolution or a dynamic kinetic resolution.

Additionally, a linear correlation was observed between the catalyst and product e.e. value, suggesting a 1:1 copper-to-ligand ratio in the enantio-discrimination complex (**Fig. S3**). X-ray crystallographic studies of **L***$Cu^{II}(OAc)_2$, synthesized by mixing **L*** with $Cu(OAc)_2$, validated the bidentate binding of the diamine catalyst with copper center (CCDC-2303333, **Fig. S4**). These results supported the involvement of a mononuclear copper species coordinated with a single chiral ligand as the active intermediate in the reaction.

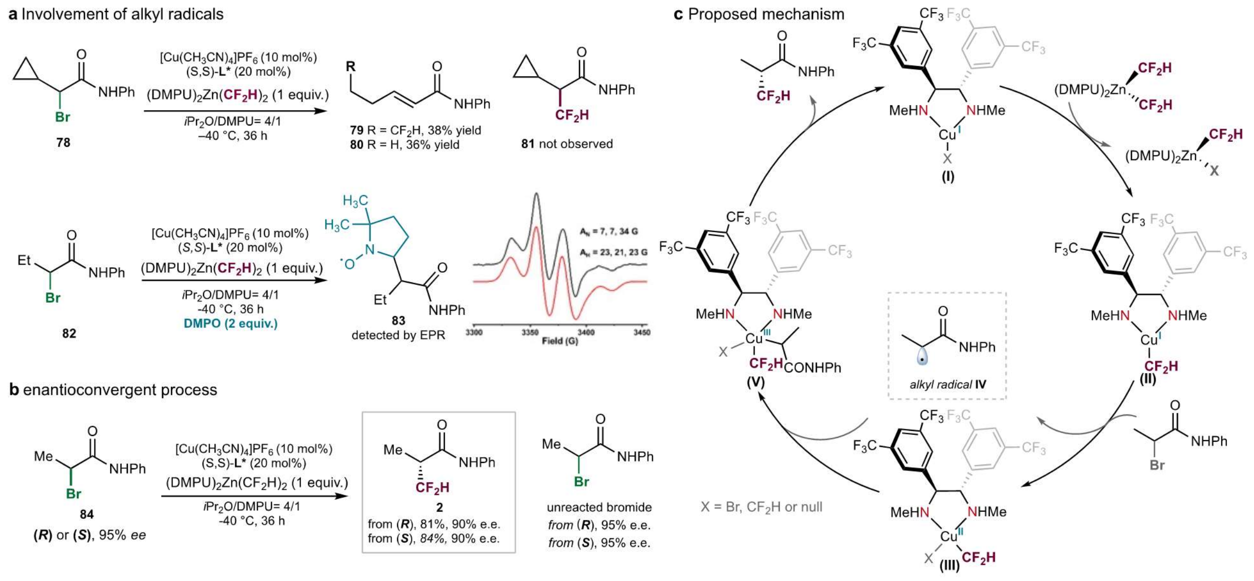

**Fig. 4** Mechanistic studies and proposed catalytic cycle. **a.** Radical clock and DMPO trapping experiments support the involvement of alkyl radical intermediates. **b.** Difluoromethylation of enantiomerically pure alkyl bromides supports a stereoablative enantioconvergent process. **c.** Proposed catalytic cycle for the enantioconvergent difluoromethylation reaction.

Based upon these experimental observations, our current hypothesis for the mechanism of this Cu-catalysed enantioselective difluoromethylation reaction is shown in **Fig. 4c**. Diamine-bound copper(I) complex **I** undergoes transmetallation with the zinc-difluoromethyl reagent to provide copper(I)–$CF_2H$ complex **II**. The reaction between complex **II** and the alkyl electrophile generates copper(II) complex **III** and an organic radical **IV**. The radical then recombines with the complex **III** to form an alkyl-copper(III)–$CF_2H$ species **V**,[51, 52] which reductively eliminates to form the difluoromethylated product and regenerate the copper(I) catalyst.[53, 54]

### Theoretical calculations

We next performed Density Functional Theory (DFT) calculations to understand the origin of enantioselectivity in this difluoromethylation process (**Fig. 5**). Previous work on copper-catalysed asymmetric functionalization of alkyl radicals indicated that the group transfer from $Cu^{II}$ intermediates to alkyl radicals is the enantio-determining step.[55] Therefore, we focused our DFT studies on the reaction between the $Cu^{II}$ intermediate [$\mathbf{L}^*Cu^{II}(CF_2H)Br$] and the alkyl radical **IV** (**Fig. 5a**). The addition to the *Re* face of the alkyl radical **IV**, which proceeded with a 1.9 kcal/mol free energy barrier at transition state **TS*R***, led to the formation of a $Cu^{III}$ intermediate **Int-*R***. In contrast, the addition from the *Si* face of **IV** occurred with a higher free energy barrier of 4.4 kcal/mol at transition state **TS*s***, resulting in the formation of the $Cu^{III}$ intermediate **Int-*S***. Both steps were exergonic, with the **Int-*R*** being 3.4 kcal/mol more stable ($\Delta G = -10.7$ kcal/mol) than its diastereomer **Int-*S*** ($\Delta G = -7.3$ kcal/mol). The C–$CF_2H$ bond formation was found to proceed via concerted reductive elimination from $Cu^{III}$ intermediates to afford either enantiomer of the difluoromethylated product. The transition state that formed the (*R*) product, **TS-RE*R***, was associated with a lower free energy ($\Delta G^‡ = 3.2$ kcal/mol) than its (*S*)-counterpart, **TS-RE*s*** ($\Delta G^‡ =$ 6.0 kcal/mol), with a difference in free energy of $\Delta\Delta G^‡ = 2.8$ kcal/mol. These computation results demonstrate that both the radical substitution and the reductive elimination steps favored the formation of the (*R*)-product, aligning well with the experimental results.

We also considered the possibility of the involvement of a bisdifluoromethyl Cu$^{II}$ intermediate, **L***Cu$^{II}$($CF_2H$)$_2$ (**Fig. S5**). Although low barriers were found to form [**L***Cu$^{III}$($CF_2H$)$_2$(alkyl)] intermediates ($\Delta G^{\ddagger} < 4$ kcal/mol), the reductive elimination of resultant Cu$^{III}$ intermediates was accompanied by high free energy barriers ($\Delta G^{\ddagger} > 29$ kcal/mol). Such high energies are unreasonable for low-temperature (–40 °C) reactions. Additionally, an alternative radical substitution pathway where the alkyl radical directly attacks the $CF_2H$ group on the Cu$^{II}$ center, without the involvement of a Cu$^{III}$ intermediate, was ruled out due to its high activation energy ($\Delta G^{\ddagger} = 23.2$ kcal/mol, **Fig. S6**).

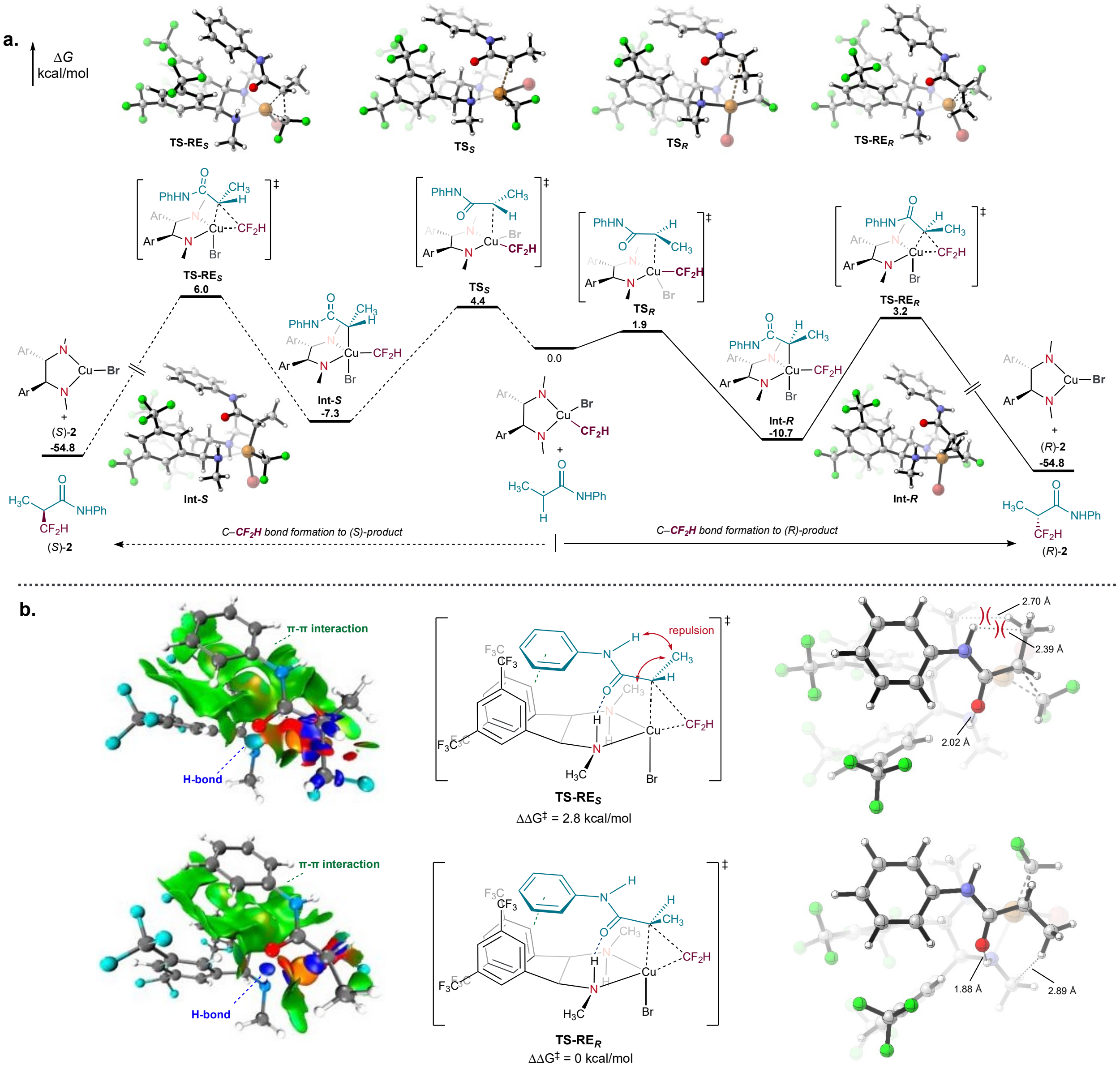

**Fig. 5** DFT calculations on the Cu-catalysed enantioconvergent difluoromethylation reaction at the B3LYP-D3BJ/def2-TZVP//B3LYP-D3BJ/def2-SVP level of theory. **a.** Free energy profile of the reactions between the Cu$^{II}$ intermediate with the alkyl radical. **b.** origin of the enantioselectivity.

To further elucidate the origin of the enantioselectivity, we examined the structures of the transition states in the C–$CF_2H$ bond-forming step. Interestingly, DFT calculations revealed that

*bis*-trifluoromethyl phenyl groups of the ligand occupied the pseudo equatorial positions, consistent with the crystal structure of $\mathbf{L}^*Cu^{II}(OAc)_2$ (**Fig. S7**). This conformation of the phenyl groups is unlikely to induce significant steric repulsion with the alkyl radical intermediate. On the other hand, the transition state structures for radical combination and reductive elimination all exhibited favorable interactions between the ligand and the substrate-derived radical (**Fig. 5a**). Interaction Region Indicator analysis[56] revealed the hydrogen bond interactions between the carbonyl group of the substrate and the NH bond of the diamine ligand (**Fig. 5b**). Furthermore, an edge-to-face π-π interaction was observed between the electron-rich phenyl groups in substrates and the electron-deficient phenyl groups in diamine catalysts. These non-covalent interactions dictated the conformation of the substrate radical during its approach to the $Cu^{II}$ intermediate and the structure of the resultant $Cu^{III}$ intermediates. Notably, the transition state that led to the *S* product (**TS-RE*s***) was destabilized due to the steric repulsion between the methyl group on the substrate and the *N*-methyl group on the ligand, along with the methyl group and N–H group on the substrate. Consequently, (*R*)-**2** was formed as the major product, consistent with the experimental results.

This stereochemical model aligned well with the experimental observations. For example, using diamine ligands with either *mono*-trifluoromethyl or non-substituted phenyl groups resulted in significantly reduced enantioselectivity (**Table S4**), likely due to weaker hydrogen-bonding and π-π interactions. Additionally, the limitation with a primary amide (**Extended Data Fig. 1**, **85**) and *N*-alkyl secondary amides (**86** and **87**) could be attributed to the lack of π-π interactions. Nonetheless, these limitations with *N*-alkyl amides could be overcome through a two-step synthesis strategy, involving the deprotection of a difluoromethylated *N*-aryl-*N*-alkyl amide **88**.

Moreover, this protocol has been successfully extended to α-bromoketones, yielding enantioenriched α-$CF_2H$ ketones with moderate to high enantioselectivity (**89** – **96,** 52-66 % yield, 76-94% e.e.). Notably, electron-rich aryl ketones (**95**) exhibited comparable or higher enantioselectivity than those with electron-neutral aryl groups (**94** and **96**), possibly due to stronger π-π interactions. Given the versatile reactivity of ketone groups, these products can be further elaborated to construct other $CF_2H$-containing stereocenters, including difluoromethylated secondary alcohols (**97** and **98,** via $NaBH_4$ reduction), esters (**99,** via Baeyer-Villiger oxidation), and tertiary alcohols (**100** and **101,** via Grignard or organolithium reagents). Finally, this method could be applied to the difluoromethylation of α-bromoesters, albeit with modest enantioselectivity, possibly due to weaker non-covalent interactions. Interestingly, using **LL***, a chiral diamine ligand containing heptafluoroisopropyl groups ($C_3F_7$), modestly improved the enantioselectivity, presumably by modulating the non-covalent interactions between the ligand and substrates.

**Conclusion:**

Overall, we report herein a Cu-catalysed approach for highly enantioselective difluoromethylation, enabling the synthesis of a broad spectrum of enantioenriched molecules containing the $CF_2H$ group. This reaction highlights the benefit of using nucleophilic fluoroalkyl reagents for the enantioselective functionalization of alkyl radical intermediates. More importantly, the successful realization of asymmetric difluoromethylation of alkyl radicals opens a new avenue for constructing stereochemical centers that contain $CF_2H$ and other fluoroalkyl groups. Finally, the manipulation of the hydrogen bonding and π-π interactions of this chiral amine ligand holds promise for inspiring the development of new asymmetric transformations.

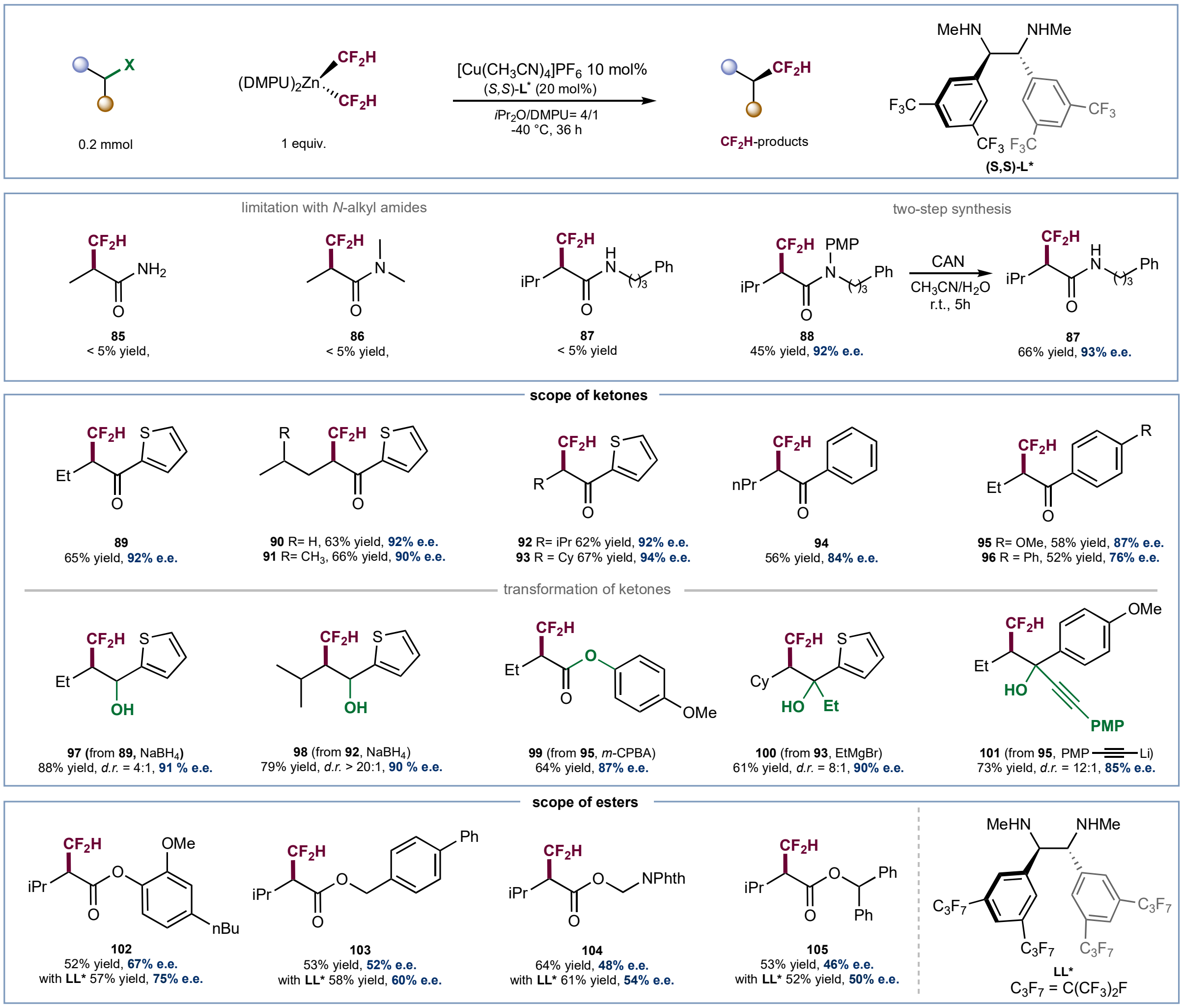

**Extended Data Fig. 1.** Other families of alkyl electrophiles investigated in this enantioconvergent difluoromethylation reaction. iPr = isopropyl, nPr = *n*-propyl, Cy = cyclohexyl, PMP = *para*-methoxyl phenyl.

### Methods:

General procedure for copper-catalysed enantioconvergent difluoromethylation of alkyl halides: In a glovebox filled with argon, an oven-dried 4 mL vial equipped with a stir bar was charged with $[Cu(CH_3CN)_4]PF_6$ (7.5 mg, 0.02 mmol, 10 mol%), ligand **L*** (20.0 mg, 0.04 mmol, 20 mol%) and anhydrous isopropyl ether (1.2 mL). The mixture was stirred at room temperature for 15 minutes, then taken out of the glovebox and cooled to –40 °C. Subsequently, a solution of the alkyl halide (0.2 mmol, 1.0 equiv.) and $(DMPU)_2Zn(CF_2H)_2$ (88 mg, 0.2 mmol, 1.0 equiv.) in DMPU (0.3 mL) was added dropwise to the reaction mixture using a syringe. After stirring for 36 h at this temperature, the reaction mixture was allowed to warm to room temperature. The difluoromethylated product was purified through column chromatography on silica gel using hexanes/EtOAc as the mobile phase. The enantiomeric excesses of the products were determined by HPLC, using AD-H, OD-H, OJ-H, IA, or IB columns.

**Acknowledgments:**

This work was supported by the National Institute of General Medical Science (R35GM146765). Mechanistic studies were supported by National Science Foundation under grant No. CHE-2237757. W.L. also. thanks the ACS Herman Frasch Foundation (926-HF22) for the financial support. NMR experiments were performed using a Bruker AVANCE NEO 400 MHz NMR spectrometer, funded by NSF-MRI grant CHE-1726092. Funding for the D8 Venture diffractometer was through NSF-MRI grant CHE-1625737.

**Author information**

Authors and Affiliations:

**Department of Chemistry, University of Cincinnati, Cincinnati, Ohio, 45221, United States**

Decai Ding, Lingfeng Yin, Jeanette A. Krause & Wei Liu

**Department of Chemistry, Purdue University, West Lafayette, Indiana 47907, United States**

Andrew T. Poore, Stephen C. Yachuw, Rachael M. Knieser & Shiliang Tian

**Department of Chemistry, National Cheng Kung University, Tainan 701, Taiwan**

Yeu-Shiuan Ho, Yu-Ho Cheng, Chi-Tien Hsieh & Mu-Jeng Cheng

Author contributions:

D.D. and W.L. designed experiments. D.D. and F.Y. performed the synthetic experiments and prepared the supplementary information. A.T.P, S.C.Y, R.M.K., and S.T. performed EPR experiments. M-J.C, Y-H.C., and C-T.H. conducted DFT calculations. J.A.K performed XRD analysis. W.L. conceived and supervised the project. W.L. wrote this manuscript with contributions from all authors.

**Competing interests:** Authors declare that they have no competing interests.

**Data availability:** The data that support the findings of this study are available within the paper, its Supplementary Information (experimental procedures, characterization data, and DFT details) and from the Cambridge Crystallographic Data Centre (https://www.ccdc.cam.ac.uk/structures; crystallographic data are available free of charge under CCDC reference numbers CCDC 2300997 and CCDC 2303333).

## Supplementary Files

This is a list of supplementary files associated with this preprint. Click to download.

- supportinginformationrevise.pdf
